# Supplementary material for: High-spatial resolution functional chemistry of nitrogen compounds in the observed UK meteorite fall Winchcombe
Source: Nat Commun. 2024 Jan 26;15:778. doi: 10.1038/s41467-024-45064-x (PMC10817942; doi:10.1038/s41467-024-45064-x)
Supplement: Supplementary file 1 — Supplementary Information [file 41467_2024_45064_MOESM1_ESM.pdf]

Supplementary Information

# High-spatial resolution functional chemistry of nitrogen compounds in the observed UK meteorite fall Winchcombe

*Christian Vollmer[1*], Demie Kepaptsoglou[2,3], Jan Leitner[4,5], Aleksander B. Mosberg[2], Khalil El Hajraoui[2], Ashley J. King[6], Charlotte L. Bays[6,7], Paul F. Schofield[6], Tohru Araki[8,9], and Quentin M. Ramasse[2,10]*

[1]Institut für Mineralogie, Universität Münster, Corrensstr. 24, 48149 Münster, Germany
*corresponding author, christian.vollmer@uni-muenster.de

[2]SuperSTEM Laboratory, Keckwick Lane, Daresbury, WA4 4AD, UK

[3]School of Physics, Engineering and Technology, University of York, Heslington, YO10 5DD, UK

[4]Institut für Geowissenschaften, Ruprecht-Karls-Universität Heidelberg, Im Neuenheimer Feld 234-236, 69120 Heidelberg, Germany

[5]Max Planck Institute for Chemistry, Particle Chemistry Department, Hahn-Meitner-Weg 1, 55128 Mainz, Germany

[6]Planetary Materials Group, Natural History Museum, London, SW7 5BD, UK

[7]Department of Earth Sciences, Royal Holloway, University of London, Egham, TW20 0EX, UK

[8]Diamond Light Source, Harwell Science and Innovation Campus, Didcot, OX11 0DE, UK

[9]National Institutes of Natural Sciences, Institute for Molecular Science, UVSOR Synchrotron Facility, 38 Nishigo-Naka, Myodaiji, Okazaki, 444-8585, Japan

[10]School of Chemical and Process Engineering and School of Physics and Astronomy, University of Leeds, Leeds LS2 9JT, UK.

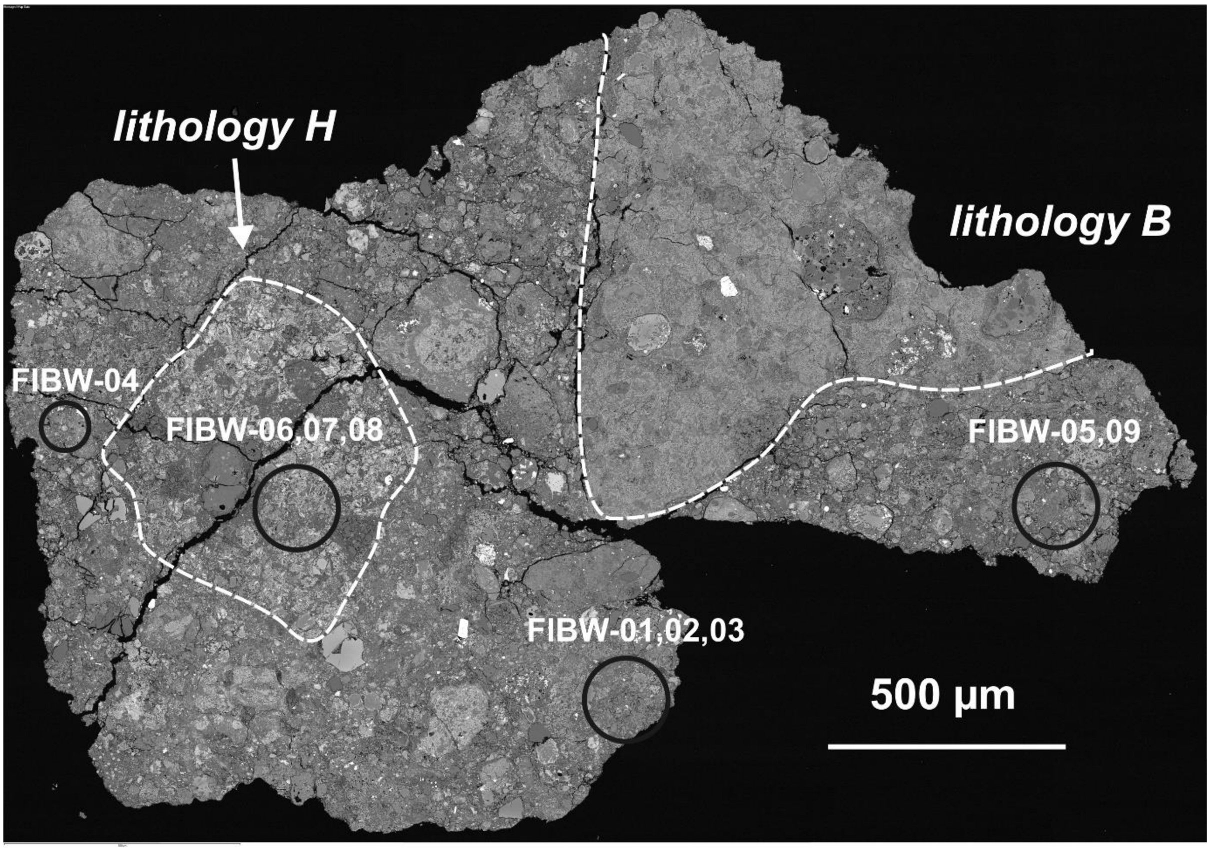


**Figure S1.** SEM-BSE (back-scattered electron) overview image of the Winchcombe fragment, showing different lithology areas and areas from which focused ion beam (FIB) thin sections were lifted out for the study.

**Table S1:** Table of N-K EELS band position assignments[19,41,42].

| Band position (eV) | Assignment |
|---|---|
| 398.8 | C=N |
| 399.8 | C≡N |
| 401.1 - 401.3 | L-alanine |
| 402.2 - 402.4 | $C-NH_x$, $CO-NR_2$, aromatic N-heterocycles |
| 403.8 | $NO_2$- |
| 405.4 | $NO_3$- |
| 405.7 - 405.9 | L-alanine, L-threonine, L-glutamine |
| 406.9 - 408.7 | N-heterocycles, nucleobases |

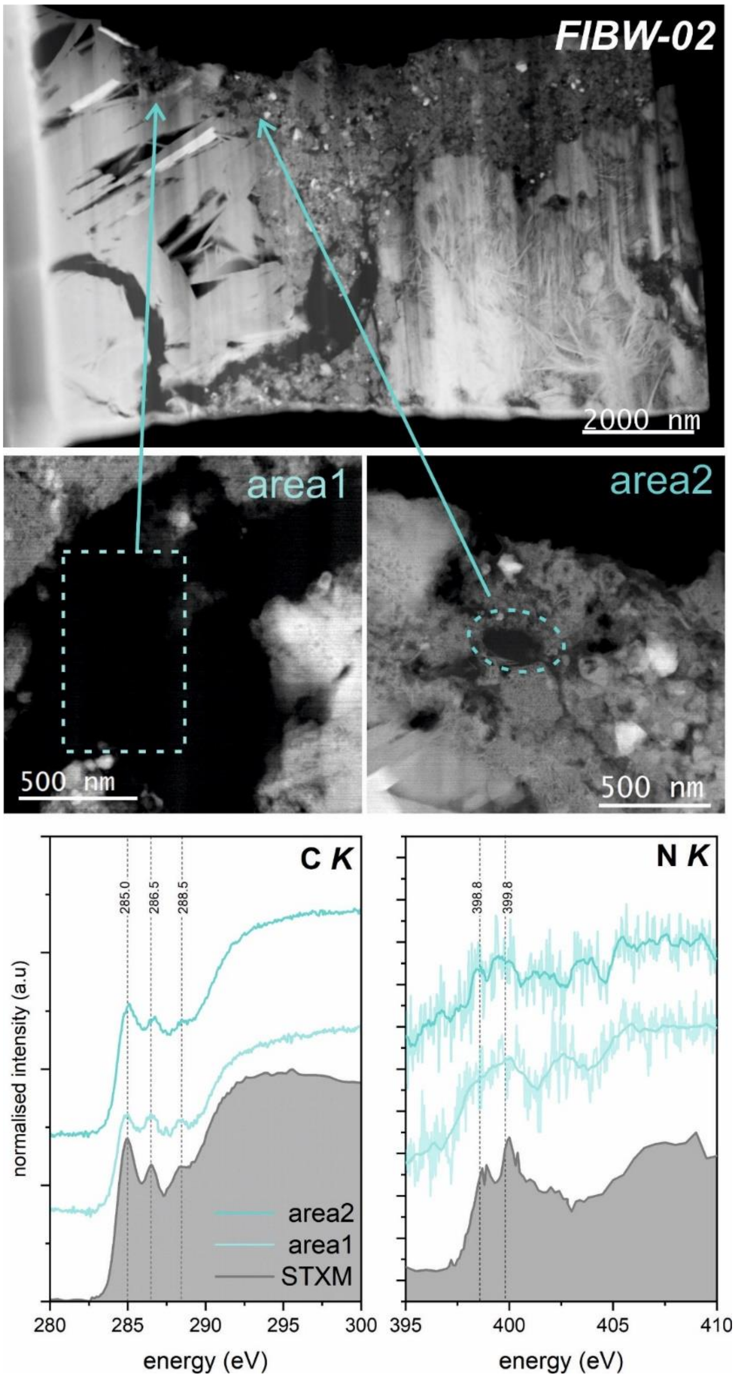


**Figure S2.** SEM-BSE overview image and STEM-HAADF images of the FIBW-02 lamella, showing areas used for EELS analysis of C-K and N-K edges plotted in the lower panels against STXM reference data acquired on the same lamella but on a larger length scale. Small nanoscale heterogeneities in the fine structure of the C-K edge are indicative of nanoscale differences in the aromaticity content.

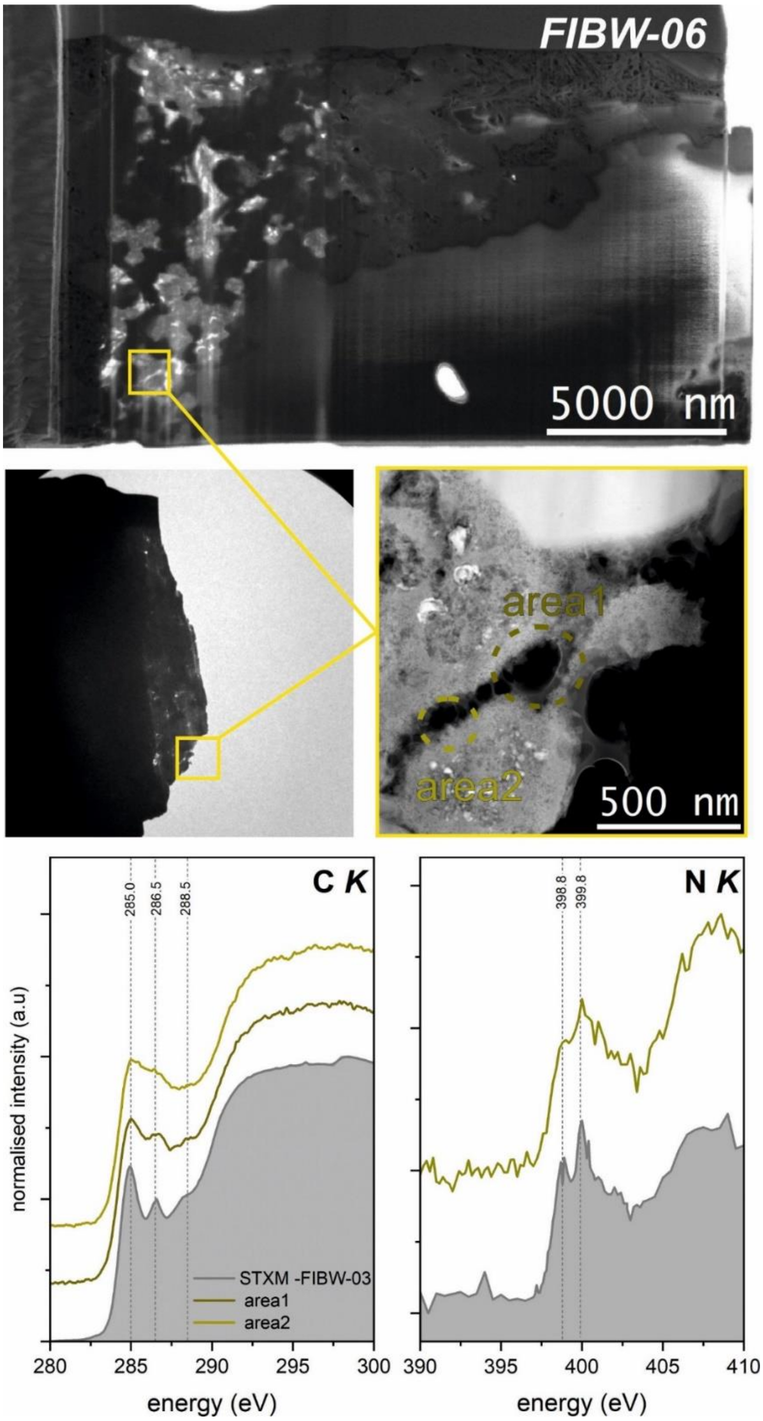


**Figure S3.** ADF-STEM and SEM (secondary electron, SE) overview images of the original FIBW-06 section used for STXM measurements. Top: overview image of the lamella, parts of which were broken during transport after STXM (middle left). The area used for C-K and N-K EELS measurements is marked. EELS data are plotted against STXM reference data acquired on the same lamella, but from possibly different positions.

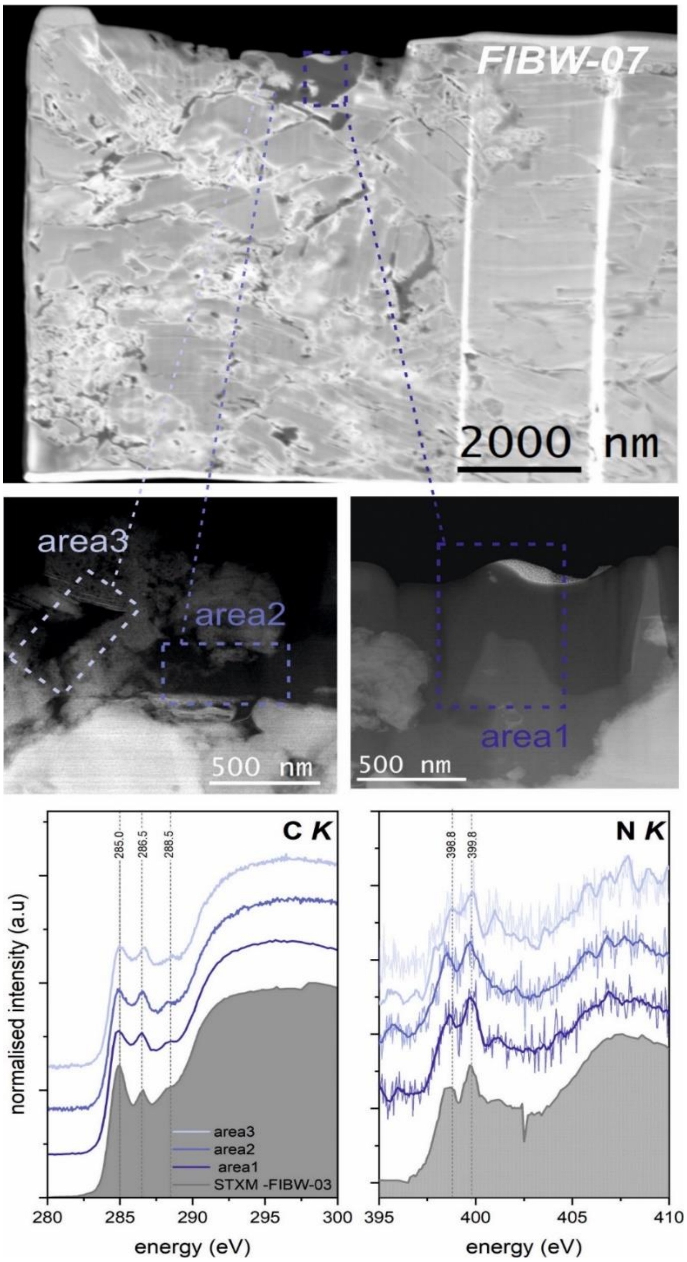


**Figure S4.** SEM-SE overview image and STEM-HAADF images of the FIBW-07 lamella, showing areas used for EELS analysis of C-K and N-K edges plotted in the lower panels against STXM reference data from the same lamella.

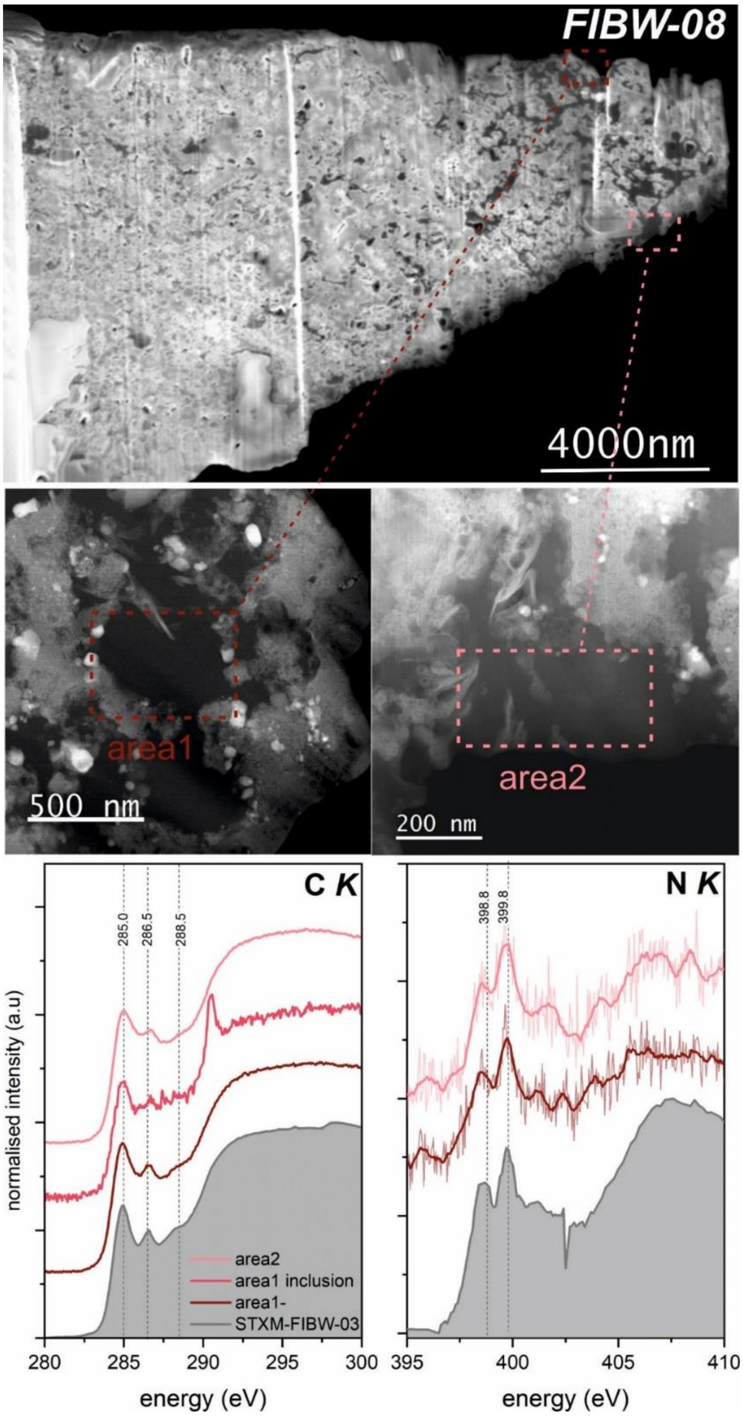


**Figure S5.** SEM-SE overview image and STEM-HAADF images of the FIBW-08 lamella, showing the areas used for EELS analysis. The C-K and N-K edges are plotted in the lower panels against STXM data from the same lamella. Small nanoscale heterogeneities in the fine structure of the C K-edge are indicative of nanoscale differences in the aromaticity content.

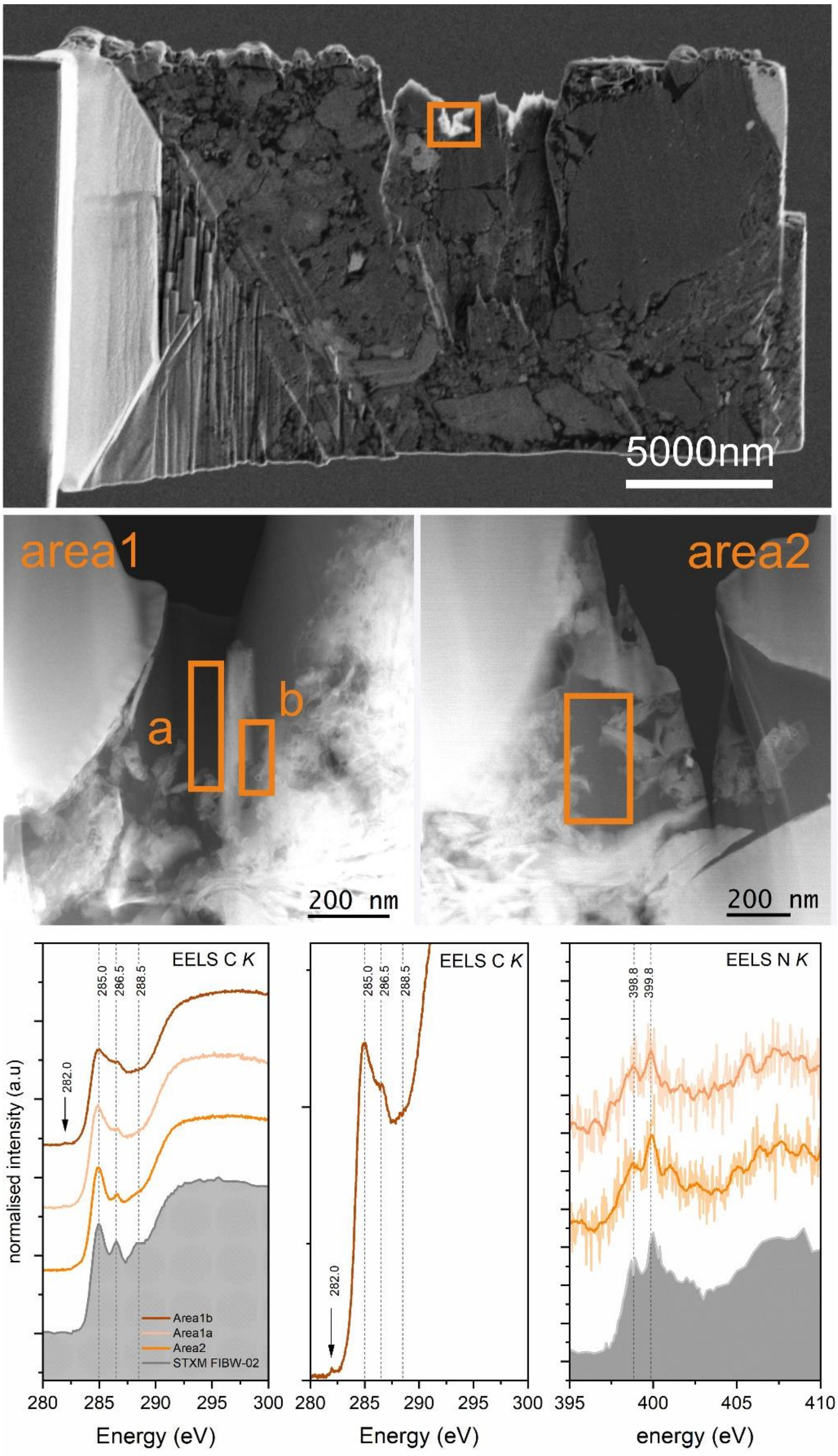


**Figure S6.** SEM-SE overview image and STEM-HAADF images of the FIBW-09 lamella, showing areas used for EELS analysis. The C-K and N-K edges are plotted in the lower panels against STXM data from the same lamella.

## EELS Data processing

*Principal Component Analysis.* EELS spectrum images were denoised using Principal Component Analysis (PCA) as implemented in the Gatan GMS3 platform, and based on work by G. Lucas et al.[40]. The use of a direct electron detector optimized for low-kV EELS provides major advantages for the analysis of complex and potentially beam sensitive OM, such as intrinsically low noise and a high detection quantum efficiency, even in low-dose conditions. The near-Poisson characteristics of the remaining noise allows efficient denoising using PCA methods[41]. A so-called Scree plot is used to determine the number of components to retain in the analysis. A typical example from our analysis is shown in Figure S7. For the reconstructions, a typical number of 10 principal components was used, over-estimated to ensure no statistically significant information is omitted from the analysis and to avoid the introduction of artefacts in the denoised data.

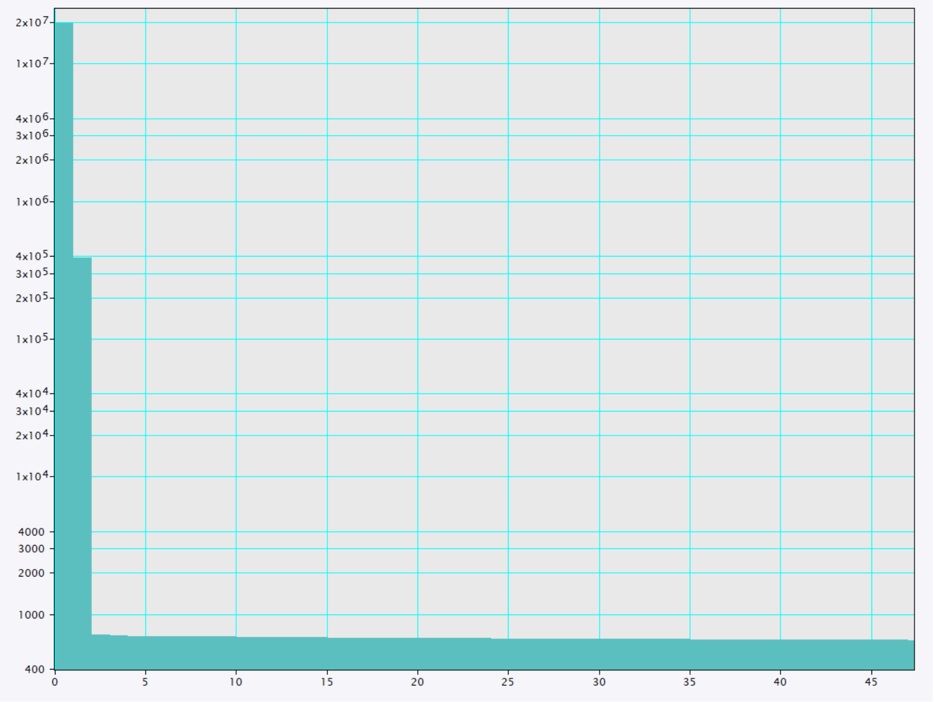


**Figure S7.** Typical Scree plot for Winchcombe N-K EELS data: the number of components used for denoising typically coincides with the point at which the Scree plot becomes linear (this is related to the nature of the noise and the central-limit theorem in the underlying statistical theory). Here, an over-estimated 10 components were used, to ensure no artefacts were introduced by the procedure.

*Smoothing procedure.* The N-K EEL spectra (data presented in Figure 8 and S2-S6) were overlaid with a smoothed line trace (using a Savitzky-Golay filter, 2$^{nd}$ order polynomial, 20-point window) as a guide to the eye. For completeness, Figure S8 compares versions of the same N-K EEL spectra as presented in Figure S6, one (black solid line) having only been denoised using PCA, and a series of smoothed spectra with different levels of smoothing (increasing numbers of points used in the window for the Savitzky-Golay kernel, as indicated). Vertical grey bands overlaid mark peaks identified in the main text, all clearly visible in the spectra, including in the non-smoothed data.

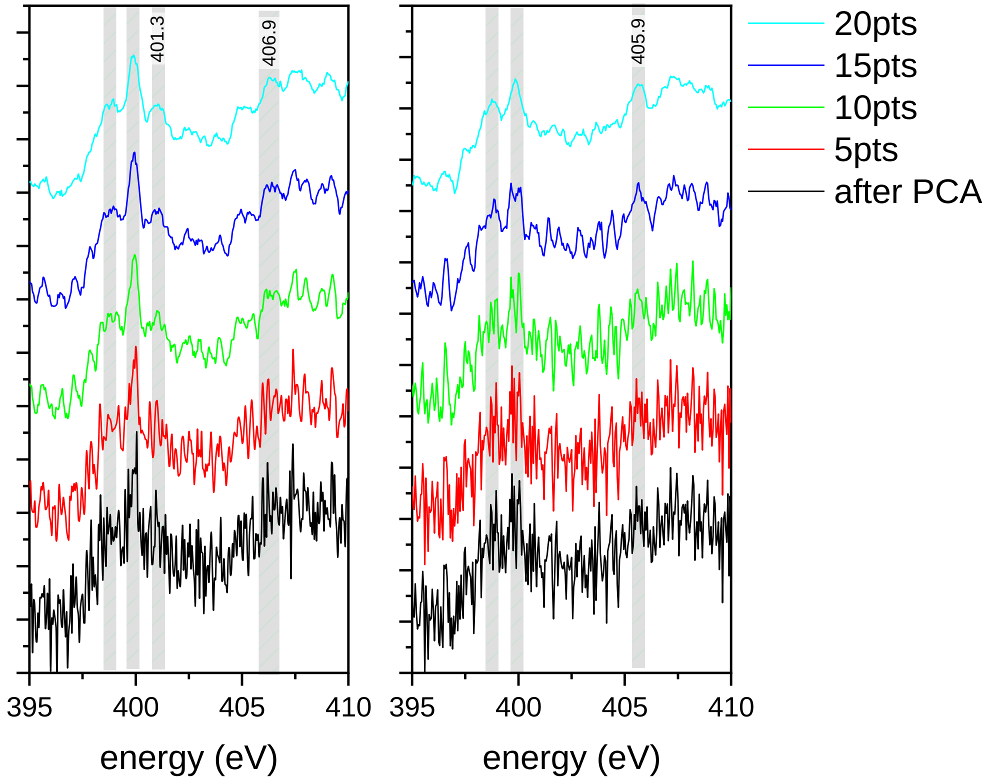


**Figure S8.** N-K EELS data from FIBW-09 with different levels of smoothing (increasing number of points in the kernel window) using the Savitzky-Golay filter, demonstrating clearly distinguishable spectral features above background, even in non-smoothed spectra.

*Blind Source Separation.* Blind Source Separation algorithms (BSS) reveal nanoscale variations of the nitrogen functional chemistry in the Winchcombe OM. This kind of multivariate statistical analysis (unsupervised machine learning) is widely used for many types of spectroscopies as it allows for the unmixing of original source signals, or 'components', from their intermixed observations[44] within complex samples. BSS also provides 'loading maps', which can be interpreted as corresponding to the spatial localization across the processed hyperspectral dataset of the corresponding 'component'. Here, the BSS analysis shows subtle but clear nanoscale variations of the N-K signal within the OM, associated with source components whose fine structure possesses distinct features, especially at energies above 400 eV. The peak at 405.9 eV, identified as corresponding to L-alanine, is particularly prominent in one of the two spectral components identified by BSS. The identification using unsupervised machine learning methods of a distinct component with spectral weight at this energy provides further validation of our assignment of this band as a recognizable feature in the N-K ELNES. Its localized variation across the loading map also provides further confidence that the observed features are not processing artefacts and confirms the complexity of the Winchcombe OM.

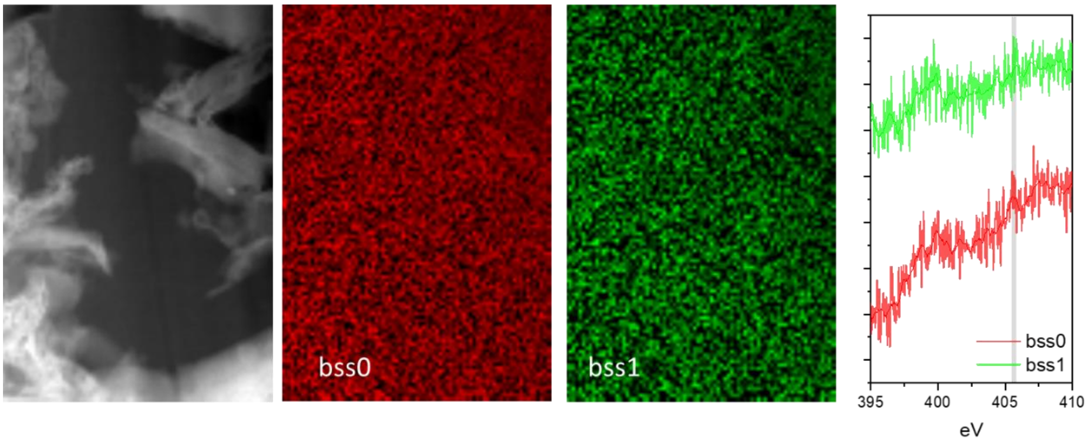


**Figure S9.** Blind Source Separation analysis of N-K EELS data from FIBW-09 showing nanoscale variations of fine structure within the OM. The first two components, labelled bss0 and bss1 (rightmost panel), show localized variations in their loading maps (middle two panels, labelled accordingly). They exhibit distinct spectral fine structure, in particular at the 405.9 eV energy (marked by a vertical grey band) identified as corresponding to the presence of L-alanine.
